# Agricultural and Food Waste: Analysis, Characterization and Extraction of Bioactive Compounds and Their Possible Utilization

**DOI:** 10.3390/foods9060817

**Published:** 2020-06-21

**Authors:** Montserrat Dueñas, Ignacio García-Estévez

**Affiliations:** Grupo de Investigación en Polifenoles, Departamento de Química Analítica, Nutrición y Bromatología, Facultad de Farmacia, Universidad de Salamanca, E37007 Salamanca, Spain; igarest@usal.es

**Keywords:** bioactive compounds, food waste, functional foods, by-products, characterization and extraction, phytochemicals, climatic change, phenolic compounds

## Abstract

The characterization and reutilization of agricultural and food waste is an important strategy to ensure the sustainable development of the agricultural and food industries. As a result, the environmental impact of these industries can be reduced, thus contributing to the fight against environmental problems, mainly to those related to a potential mitigation of climatic change. This Special Issue includes five papers that reported important findings from research activities related to the reutilization of by-products from food processing industries, which help to increase the knowledge in this field.

The food processing industries produce millions of tons of losses and waste during processing, which is becoming a grave economic, environmental and nutritional problem. Fruit, vegetable and food industrial solid waste includes several products that are released in food production during cleaning, processing, cooking, and/or packaging. These wastes can be an important source of bioactive compounds since, in their composition, important levels of phenolic compounds, dietary fibers, polysaccharides, vitamins, carotenoids, pigments and oils, among others, can be found. These compounds, in turn, are closely associated with beneficial effects in human health. Thus, these by-products can be exploited again in the food industry to develop functional ingredients and/or new foods or natural additives or, in other industries, such as the pharmaceutical, agricultural or chemical industries, to obtain cosmetic products, fertilizers or animal feed, among other things. Therefore, the characterization and reutilization of these by-products is important to ensure the sustainable development of the food industry and reduce its environmental impact, which would contribute to the fight against environmental problems, mainly to a potential mitigation of climatic change.

Among the submitted works, five papers have been selected to be included in this Special Issue. The study performed by Mislata and co-workers assesses the levels and identities of the aromatic and bioactive compounds, which, in turn, can be related to beneficial health effects, that can be found in the by-products of cork stopper production [1]. Thus, these authors reported the potential added value of the waste of that industry. To be precise, three different cork granulates that can be used to produce cork stoppers, which differed in their size, along with the corresponding cork stoppers, were analyzed in order to determine the phenolic and aromatic compositions, as well as the antioxidant activity of the corresponding extracts. The results indicated that several aromatic compounds with industrial interest can be extracted mainly from the cork by-products, among them, vanillins and volatile phenols such as 4-vinylguaiacol. The cork by-products also showed important concentrations of phenolic compounds, gallic and protocatechuic acids being the most important phenolic compounds extracted. These phenolic compounds are related by these authors to the high antioxidant activity determined in the extracts obtained from cork by-products, although aromatic compounds such as vanillin can also be responsible for that activity. Altogether, these authors reported the high potential of cork by-products and cork stoppers, but mainly the former, to be reused as flavoring agents and antioxidants in the food industry.

The study carried out by Jiang et al. is also included in this Special Issue. In this paper, an innovative methodology for the extraction and purification of ovalbumin from salted egg whites, obtained as by-products of the industrial obtention of salted egg yolks, is outlined [2]. This represents an important step for the reutilization of these by-products that are usually discarded as waste because of the difficulty in treatment and that can cause important environmental problems due to their salt content. The new methodology comprises an aqueous two-phase flotation that allows the separation of ovalbumin from the food by-products. This process, described as simple, inexpensive and efficient, was developed and optimized by using a response surface method experiment, and it allowed the authors to obtain a purified ovalbumin extract, which was characterized by means of different techniques such as SDS-PAGE, RP-HPLC, nano-LC-ESI-MS/MS, UV, fluorescence and FT-IR spectroscopy. This characterization reveals the high purity of the ovalbumin obtained, whose structure and properties in oil binding capacity, viscosity, emulsibility and foam capacity did not show differences with the corresponding standard. Thus, this new methodology can be considered as a sustainable and effective way for the utilization of salted egg whites to reduce environmental pollution.

This Special Issue also includes the study performed by F. Correddu et al. [3], in which the proximate composition and phenolic compounds content, as well as the antioxidant activity of *Myrtus communis* berries obtained as waste from myrtle-liqueur production, have been studied. These analyses were also carried out in the different parts of berries: seeds and pericarps. These berries are typically used to elaborate a sweet myrtle-liqueur by their hydroalcoholic infusion for at least two weeks. The results obtained by these authors demonstrated that exhausted myrtle berries presented a high concentration of carbohydrates, proteins and lipids, showing that the seeds have higher levels of hemicellulose, cellulose and lipids than pericarps and whole berries. The lipid fraction showed a high concentration of polyunsaturated fatty acids, linoleic acid being the most important fatty acid in seeds and whole exhausted myrtle berries. With regard to the phenolic profile, hydroxybenzoic and hydroxycinnamic acids and flavonols such as quercetin, isorhamnetin and kaempferol were identified in these berries. Ellagic acid was the one presented in the highest levels, followed by gallic acid. Among the flavonols, quercetin aglycon and quercetin 3-*O*-rutinoside were the most abundant flavonoids. This study showed that the seeds presented the highest total phenolic compounds concentration and, as a consequence, the highest antioxidant activity. Hence, this study suggests the importance of this by-product with multiple industrial applications, as a food ingredient or in animal feed formulations.

The study carried out by Vella et al. [4] is focused on total phenolic compounds, ortho-diphenol, flavonoid and tannin contents and antioxidant activity of peels and seeds from Cantaloupe melon. The Cantaloupe melon is the most cultivated variety in Italy and it is characterized by its high content of vitamins A and C and minerals such as potassium and magnesium [5]. The melon processing industries produce great quantities of waste during processing, mainly peel and seeds. These by-products could be exploited in order to minimize economic and environmental problems. The results reported suggest that peel and seeds of Cantaloupe melon are promising sources of natural phytochemicals, which could be related to their high polyphenol and tannin concentrations, mainly in the case of peels. These investigations would be useful for the food industry to develop new nutraceuticals, as well as to reduce waste volumes, thus contributing to the sustainable management of waste that involves environmental and economic costs.

S. Silbir and Y. Goksungur [6] have carried out studies on the re-utilization of brewery waste hydrolysate with the aim of obtaining natural red pigments by *Monascus purpureus* CMU001, used in the fermentation of brewer’s spent grain. This study was mainly focused on the chemical, structural and elemental characterization of this by-product using different techniques such as Fourier-transform infrared spectroscopy (FT-IR) and X-Ray Photoelectron Spectroscopy (XPS). The obtained results show that the brewer’s spent grain was constituted mainly by lignin, hemicellulose, cellulose and protein, whose presence was confirmed by infrared spectrum (FT-IR). The XPS analysis indicated the presence of carbon, oxygen, nitrogen and phosphorus. These authors also reported the optimization of the fermentation process to obtain the maximum natural red pigment concentration by *Monasuc purpureus*, reaching the highest biomass concentration at 7 days of fermentation, after which it declined. Therefore, this study suggests an important alternative for using this waste, produced in great quantities in breweries, and thus, to obtain natural pigments, which present multiple beneficial properties for health.

We are pleased to present this Special Issue, which includes five papers that highlight the most important of the research activities in the field of the reutilization of by-products from food processing industries, which could contribute to the mitigation of environmental problems, mainly in terms of their climatic change potential. We are very grateful to the authors who have shared their scientific knowledge and experience through their contribution to this Special Issue. We sincerely hope that the readers will find this Special Issue interesting and informative.
